# Association between tobacco smoke exposure and constipation among American adults: a National Health and Nutrition Examination Survey

**DOI:** 10.3389/fpubh.2025.1502341

**Published:** 2025-03-04

**Authors:** Guoce Cui, Xia Jiao, Zhenbiao Wang, Zhuhui Zhang

**Affiliations:** ^1^Department of Traditional Chinese Medicine Surgery, Beijing Shijitan Hospital, Capital Medical University, Beijing, China; ^2^Department of Anorectology, Guang'anmen Hospital, China Academy of Traditional Chinese Medicine, Beijing, China

**Keywords:** constipation, tobacco, smoke exposure, NHANES, serum cotinine

## Abstract

**Background:**

Studies have shown that there is a relationship between smoke exposure and constipation. However, there are limited data on the associations between constipation and smoking or serum cotinine levels, a metabolite of nicotine commonly used as a marker of tobacco exposure.

**Methods:**

This study thoroughly examined the cross-sectional data from the National Health and Nutrition Examination Survey (NHANES) from 2005 to 2010. We applied multivariate logistic regression models to assess the association between tobacco smoking status, serum cotinine levels, and constipation.

**Results:**

In this cross-sectional study, 11,651 participants were included. The average age of the participants was 48.8 ± 17.9 years. After controlling for covariates, there was no significant relationship between serum cotinine levels, smoking status, and constipation risk. According to the categorical analysis, the incidence of constipation was 36% higher in participants with serum cotinine levels between 0.05 and 2.99 ng/mL (model 1: OR = 1.45 [1.13–1.85]; model 2: OR = 1.44 [1.12–1.83]; and model 3: OR = 1.36 [1.06–1.74]; *p* < 0.05) after adjusting for covariates by using the lowest levels of serum cotinine (<0.05 ng/mL) as a reference. In the non-smokers, serum cotinine levels were linearly and positively associated with the risk of constipation (*p* > 0.05), and the relationship between smoke exposure and constipation remained relatively stable in across all subgroups.

**Conclusion:**

The study suggests that serum cotinine levels have a promoting effect on stool frequency-related constipation in non-smokers. Therefore, avoiding passive smoking as much as possible may reduce the effect of smoke exposure on constipation and serve as a preventive measure for treatment.

## Introduction

Chronic constipation is a gastrointestinal disorder marked by unsatisfactory bowel movements. Symptoms include difficulty defecating, infrequent bowel movements, painful bowel movements, hard stools, and a feeling of incomplete defecation. Chronic constipation affects 27% of the population in Western countries, with a higher prevalence among women, older individuals, and those with lower socio-economic status (1). Millions of people around the globe suffer from constipation, which is considered a significant public health issue. Aside from the economic costs, constipation significantly affects the quality of life of patients, both mentally and physically (2). The risks of constipation are related to factors such as the living environment, dietary habits, and unhealthy behaviors (3). However, few studies have evaluated the prognostic effect of tobacco smoke exposure on constipation in American adults, especially in large sample sizes.

Smoking, as well as passive inhalation of environmental tobacco smoke (4–6), damages many organs in the body, leading to digestive diseases, such as constipation (7). Smoke exposure may cause intestinal symptoms by regulating intestinal flora (8, 9). Cotinine, a byproduct of nicotine, is commonly utilized as a marker for tobacco smoke exposure within the past 72 h. It is considered a reliable and sensitive marker of such exposure (10–12).

However, there is a lack of clinical studies on the relationship between tobacco smoke exposure and the risk of constipation. This study will reflect the relationship between tobacco smoke exposure and chronic constipation from multiple perspectives based on smoking status and serum cotinine levels. This cross-sectional study involving a vast national sample of 11,651 American adults as participants aimed to show the relationship between serum cotinine levels, smoking behavior, and the occurrence of constipation by analyzing the NHANES data from 2005 to 2010. The findings of our study demonstrate epidemiological evidence to support further investigation of the relationship between exposure to tobacco smoke and constipation.

## Materials and methods

### Study population and design

The NHANES data from 2005 to 2010 (13) were used for this cross-sectional study conducted by the Centers for Disease Control and Prevention. Using a survey conducted in stages with varying levels of sampling probabilities, the NHANES project evaluated the health and nutritional situation of non-institutionalized Americans (14). Using a mobile examination center (MEC), an integrated system of home visits, laboratory tests, and screenings is applied to gather demographic and health information for the NHANES. Participants in the NHANES provided written informed consent before participating in the study, which was approved by the Ethics Review Committee of the National Center for Health Statistics (NCHS). It was unnecessary to obtain additional approval from the Institutional Review Board for the secondary analysis (15). Data from the NHANES can be accessed by visiting the NHANES website.^1^ The research involved individuals aged 20 years and more who had completed the questionnaire. Individuals with missing serum cotinine levels, smoking status, or constipation were excluded.

### Assessment of tobacco exposure

The term “never smoker” is applied to smokers aged ≥20 who claim to have never smoked more than 100 cigarettes throughout their existence during the household interview. Individuals who were former smokers had smoked over 100 cigarettes before quitting, while those who are currently smoking smoke regularly. A measure of secondhand smoke exposure and active smoking is cotinine, nicotine’s primary metabolite (16). Isotope dilution-high-performance liquid chromatography/atmospheric pressure chemical ionization tandem mass spectrometry was used to measure serum cotinine levels (17). Previous investigations (18) considered those below the lower detection threshold unexposed. To represent smoking exposure, we created cotinine categories using the newly recommended cutoff point of 3 ng/mL, as suggested by a previous study (19), to differentiate smokers from non-smokers. Cotinine levels were categorized into three groups: cotinine <0.05 ng/mL, cotinine 0.05–2.99 ng/mL, and cotinine ≥3 ng/mL.

### Definition of constipation

To diagnose constipation, stool frequency and consistency were considered important factors based on the Rome IV criteria (20) and prior NHANES studies (21–26). Our study defined chronic constipation primarily based on stool frequency, as assessed in the NHANES Intestinal Health Questionnaire. By asking about the frequency of stool habits, the frequency of bowel movements was determined: “How many times per week do you usually have a bowel movement?” Constipation (<3 stools/week), diarrhea (≥21 stools/week), and normal were classified as the results. Additionally, a card with colored photos and clarifications of the seven Bristol Stool Form Scale (BSFS) types (Type 1 to Type 7) was shown to the participants, and they were asked to indicate which type of stool corresponded to their most common stool type. Constipation is characterized by hard lumpy or lumpy stools of BSFS Type 1 (like nuts) or Type 2 (like sausages), and diarrhea is characterized by soft fragments with uneven borders and mushy stool types 6 or 7 (watery with no solid chunks). The remaining subjects were considered to have normal stool habits.

### Covariates

In accordance with the literature, a variety of potential covariates were assessed (3, 20, 21, 23, 26), including age, sex, ethnicity, education level, marital status, physical activity, body mass index (BMI), dietary factors [calorie consumption, protein consumption, carbohydrate consumption, sugar consumption, fiber consumption, fat consumption, caffeine consumption, alcohol consumption, water (moisture) consumption, and dietary supplements], and previous diseases (diabetes, stroke, and coronary heart disease). Ethnicity was categorized as non-Hispanic white, non-Hispanic black, Mexican American, or other ethnicities. Educational attainment was ranked as less than 9 years, 9–12 years, and more than 12 years. Marital status was classified as married, living with a partner, or living alone. Physical activity was defined as participating in high-intensity sports, exercise, or leisure activities, such as running or basketball, that cause a significant increase in breathing or heart rate for a continuous period of at least 10 min. According to a standardized technique, BMI was calculated using height and weight. Participants’ 24-h nutritional information was obtained via a dietary recall interview conducted before an interview at an MEC. This included data on total dietary calories, protein, carbohydrates, sugar, fiber, fat, caffeine, alcohol, and water (moisture). Supplement usage over the past month was determined through a question about nutritional supplements and medications. According to the questionnaire, previous diseases such as diabetes, stroke, and coronary heart disease, are based on whether a doctor had previously diagnosed the condition.

### Statistical analysis

The means (standard deviations) and medians (interquartile ranges) were calculated and compared for continuous variables using either the Student’s t-test for normal distribution or the Mann–Whitney U-test for non-normal distribution. Categorical variables were displayed as absolute values in percentages and analyzed using the chi-square test. Cotinine levels were log2-transformed into a normal distribution as continuous variables.

We undertook several diagnostic tests to examine basic logistic regression assumptions (28), and then three consecutive multivariate logistic regression models were used to examine the relationship between tobacco exposure and the occurrence of constipation. Model 1 was adjusted for age, sex, ethnicity (non-Hispanic white, non-Hispanic black, Mexican American, or other ethnicities), educational attainment (less than 9 years, 9–12 years, and more than 12 years), physical activity, marital status (married, living with a partner, or living alone), and BMI (continuous). Model 2 was based on model 1 and added previous diseases (diabetes, stroke, and coronary heart disease). Model 3 was adjusted for model 2 and included additional adjustments for dietary factors [calorie consumption, protein consumption, carbohydrate consumption, sugar consumption, fiber consumption, fat consumption, caffeine consumption, alcohol consumption, and water (moisture) consumption] and dietary supplements.

In addition, restricted cubic spline (RCS) regression was used to examine linearity and assess the dose–response curve between log2-transformed serum cotinine levels and constipation risk after adjusting variables in model 3. Subgroup analyses were stratified by relevant confounders based on their association with the outcomes of interest. The analysis was conducted using R statistical software (Version 4.2.2, The R Foundation)^2^ and Free Statistics analysis platform (Version 1.8, Beijing, China) (27, 29). All participants were involved in a descriptive study. Using two-tailed testing, we determined that a *p*-value of 0.05 was significant.

## Results

### Baseline characteristics of participants

**Figure 1:**
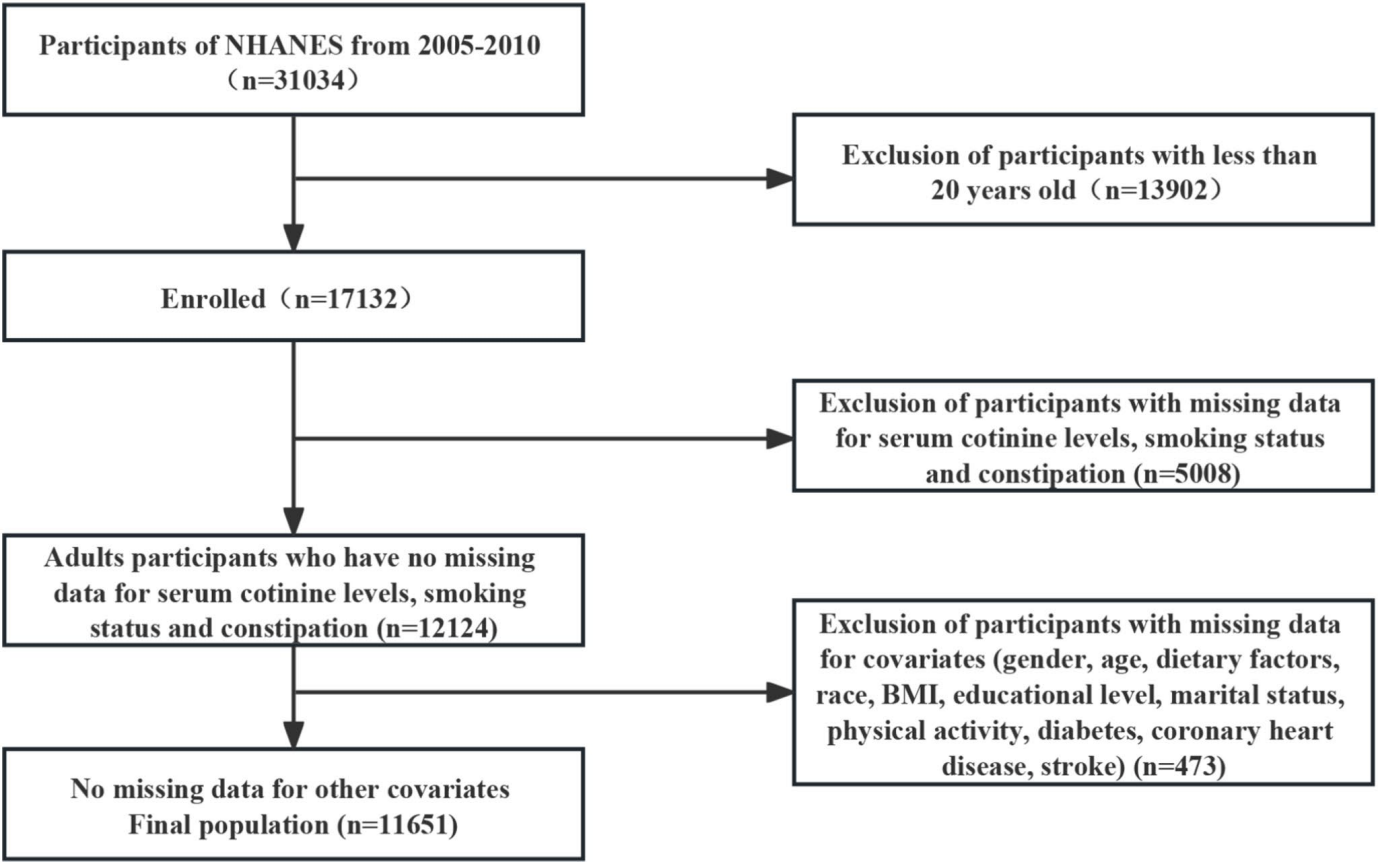
Flowchart of participant selection.

This study selected 31,034 potential participants from the NHANES (2005–2010). Excluded from the study were individuals under the age of 20 (*n* = 13,902) and those who lacked information on serum cotinine levels, smoking status, and constipation (*n* = 5,008). We included 12,124 participants after excluding those with missing covariate data (*n* = 473) in our analysis. The flowchart in Figure 1 illustrates the exclusion criteria. The baseline characteristics of the research individuals are shown in Table 1 according to the NHANES 2005–2010. In this sample, a total of 3,173 (27.2%) participants had cotinine levels ≥3 ng/mL, and 2,550 (21.9%) individuals in the reference group were current smokers. Compared to women, men have significantly higher cotinine levels.

**Table 1:** Baseline characteristics of participants.

Covariates	Total (*n* = 11,651)	Cotinine category, %			*p*-value	Self-reported smoking status, %			*p*-value
		<0.05 ng/mL (*n* = 5,607)	0.05–2.99 ng/mL (*n* = 2,871)	≥3 ng/mL (*n* = 3,173)		Never (*n* = 6,187)	Former (*n* = 2,914)	Current (*n* = 2,550)	
Sex, n (%)					<0.001				<0.001
Male	5,727 (49.2)	2,383 (42.5)	1,438 (50.1)	1,906 (60.1)		2,525 (40.8)	1,750 (60.1)	1,452 (56.9)	
Female	5,924 (50.8)	3,224 (57.5)	1,433 (49.9)	1,267 (39.9)		3,662 (59.2)	1,164 (39.9)	1,098 (43.1)	
Age (years)	48.8 ± 17.9	52.1 ± 18.1	47.7 ± 18.4	43.9 ± 15.9	<0.001	47.0 ± 18.1	57.1 ± 17.1	43.6 ± 15.2	<0.001
Ethnicity, n (%)					<0.001				<0.001
Non-Hispanic white	5,933 (50.9)	2,858 (51)	1,340 (46.7)	1,735 (54.7)		2,769 (44.8)	1,782 (61.2)	1,382 (54.2)	
Non-Hispanic black	2,193 (18.8)	720 (12.8)	731 (25.5)	742 (23.4)		1,234 (19.9)	417 (14.3)	542 (21.3)	
Mexican American	2,083 (17.9)	1,220 (21.8)	482 (16.8)	381 (12)		1,290 (20.9)	440 (15.1)	353 (13.8)	
Others	1,442 (12.4)	809 (14.4)	318 (11.1)	315 (9.9)		894 (14.4)	275 (9.4)	273 (10.7)	
Education level (year), n (%)					<0.001				<0.001
<9	1,254 (10.8)	650 (11.6)	315 (11)	289 (9.1)		678 (11)	326 (11.2)	250 (9.8)	
9–12	4,602 (39.5)	1,681 (30)	1,220 (42.5)	1,701 (53.6)		2,124 (34.3)	1,074 (36.9)	1,404 (55.1)	
>12	5,795 (49.7)	3,276 (58.4)	1,336 (46.5)	1,183 (37.3)		3,385 (54.7)	1,514 (52)	896 (35.1)	
Marital status, n (%)					<0.001				<0.001
Married or living with a partner	7,238 (62.1)	3,872 (69.1)	1,638 (57.1)	1,728 (54.5)		3,863 (62.4)	1,984 (68.1)	1,391 (54.5)	
Living alone	4,413 (37.9)	1,735 (30.9)	1,233 (42.9)	1,445 (45.5)		2,324 (37.6)	930 (31.9)	1,159 (45.5)	
Physical activity, n (%)	2,736 (23.5)	1,360 (24.3)	715 (24.9)	661 (20.8)	<0.001	1,693 (27.4)	554 (19)	489 (19.2)	<0.001
BMI (kg/m^2^)	28.8 ± 6.5	28.8 ± 6.2	29.9 ± 7.1	27.9 ± 6.5	<0.001	29.1 ± 6.7	29.3 ± 6.2	27.6 ± 6.4	<0.001
Dietary factors									
Energy (kcal)	2,113.4 ± 991.5	1,989.0 ± 850.3	2,103.2 ± 935.3	2,342.5 ± 1208.9	<0.001	2032.9 ± 910.9	2084.7 ± 898.4	2341.4 ± 1220.0	<0.001
Protein (gm)	81.0 ± 42.2	78.2 ± 37.9	80.9 ± 40.0	86.3 ± 50.0	<0.001	79.2 ± 40.1	80.7 ± 37.6	85.8 ± 50.8	<0.001
Carbohydrate (gm)	257.5 ± 125.9	247.2 ± 109.5	255.3 ± 119.3	277.7 ± 153.5	<0.001	252.7 ± 116.0	248.3 ± 113.1	279.6 ± 156.6	<0.001
Sugar (gm)	101.3 (63.3,150.5)	97.1 (62.5,139.4)	100.6 (63.1,151.1)	111.7 (66.4,172.3)	<0.001	100.8 (64.1,147.2)	95.2 (60.4,139.3)	112.6 (66.1,175.4)	<0.001
Fiber (gm)	16.1 ± 9.7	17.6 ± 9.8	15.2 ± 8.8	14.3 ± 9.6	<0.001	16.4 ± 9.5	16.9 ± 9.6	14.3 ± 9.8	<0.001
Coffee (mg)	101.0 (15.0, 223.0)	90.0 (10.0,201.0)	87.0 (10.0, 199.0)	149.0 (44.0, 313.0)	<0.001	73.0 (6.0, 168.0)	134.0 (39.0, 251.0)	166.0 (56.0, 344.8)	<0.001
Alcohol (gm)	0.0 (0.0, 0.2)	0.0 (0.0, 0.0)	0.0 (0.0, 0.0)	0.0 (0.0, 21.2)	<0.001	0.0 (0.0, 0.0)	0.0 (0.0, 11.4)	0.0 (0.0, 19.4)	<0.001
Fat (gm)	70.4 (47.1,101.1)	67.5 (44.9, 95.7)	71.1 (47.8, 102.4)	76.5 (51.3, 110.5)	<0.001	67.9 (45.2, 96.6)	72.8 (48.9, 102.2)	75.6 (51.0, 110.5)	<0.001
Moisture (gm)	2881.9 ± 1452.3	2737.2 ± 1289.8	2781.6 ± 1369.3	3228.5 ± 1715.5	<0.001	2733.6 ± 1342.5	2864.2 ± 1364.6	3262.2 ± 1714.3	<0.001
Dietary supplements, n (%)	5,740 (49.3)	3,311 (59.1)	1,336 (46.5)	1,093 (34.4)	<0.001	3,009 (48.6)	1,200 (41.2)	1702 (66.7)	<0.001
Diabetes, n (%)	1,208 (10.4)	628 (11.2)	320 (11.1)	260 (8.2)	<0.001	605 (9.8)	416 (14.3)	187 (7.3)	<0.001
Coronary heart disease, n (%)	462 (4.0)	234 (4.2)	110 (3.8)	118 (3.7)	0.528	163 (2.6)	220 (7.5)	79 (3.1)	<0.001
Stroke, n (%)	373 (3.2)	182 (3.2)	92 (3.2)	99 (3.1)	0.949	139 (2.2)	149 (5.1)	85 (3.3)	<0.001
Constipation (stool frequency), n (%)	440 (3.8)	162 (2.9)	138 (4.8)	140 (4.4)	<0.001	230 (3.7)	88 (3)	122 (4.8)	0.003
Constipation (stool consistency), n (%)	973 (8.4)	476 (8.5)	269 (9.4)	228 (7.2)	0.008	582 (9.4)	205 (7)	186 (7.3)	<0.001

BMI, body mass index.

The average age of the study population was 48.8 ± 17.9 years. In this study, 3,173 (27.2%) participants had cotinine levels above 3 ng/mL, 2,871 (24.6%) participants had cotinine levels between 0.05 and 2.99 ng/mL, and 5,607 (48.1%) participants had cotinine levels below 0.05 ng/mL. There were 2,550 (21.9%) current smokers, 2,914 (25.0%) former smokers, and 6,187 (53.1%) non-smokers among the participants who self-reported their smoking status. Self-reported current smokers and individuals with cotinine levels >3 ng/mL were more likely to be younger, male, and non-Hispanic white, have 9–12 years educational attainment, married or living with a partner, have less physical activity and BMI, have higher daily dietary intakes of energy, protein, carbohydrate, sugar, fat, caffeine, alcohol, moisture, lower fiber, and dietary supplements, and have a lower incidence of diabetes, coronary heart disease, and stroke than those with a serum cotinine level < 0.05 ng/ mL and those who were self-proclaimed non-smokers. We found that 3.8% of American adults experienced constipation based on bowel frequency.

### Associations between serum cotinine levels and smoking status with constipation

Based on the univariate analysis, gender, age, ethnicity, marital status, physical activity, BMI, stroke, and dietary intakes, such as total energy, protein, carbohydrate, fiber, fat, alcohol, moisture, and dietary supplements were associated with constipation (Table 2). Using both continuous and categorical perspectives, the multivariate logistic regression analysis illustrates the relationship between serum cotinine levels, smoking status, and the risk of constipation (Table 3 and Supplementary Table 2). In contrast to our previous assumptions, after controlling for covariates, there was no significant relationship between the cotinine levels transformed by log2 and constipation risk. Interestingly, according to the categorical analysis, a significant effect of serum cotinine on stool frequency-related constipation is observed in a population with cotinine levels between 0.05 and 2.99 ng/mL. The incidence of constipation was 36% higher in participants with serum cotinine levels between 0.05 and 2.99 ng/mL (model 1: OR = 1.45 [1.13–1.85]; model 2: OR = 1.44 [1.12–1.83]; model 3: OR = 1.36 [1.06–1.74]; *p* < 0.05) after adjusting for covariates using the lowest levels of serum cotinine (<0.05 ng/mL) as a reference.

**Table 2:** Association of covariates and constipation.

Variable	OR_95 CI%	*p*-value
Sex, n (%)		
Male	1 (reference)	
Female	3.5192 (2.8029 ~ 4.4185)	<0.001
Age (years)	0.9848 (0.9794 ~ 0.9903)	<0.001
Ethnicity, n (%)		
Non-Hispanic white	1 (reference)	
Non-Hispanic black	2.0042 (1.6086 ~ 2.497)	<0.001
Mexican American	0.644 (0.4656 ~ 0.8907)	0.0078
Others	1.0031 (0.7301 ~ 1.3783)	0.9846
Education level (year), n (%)		
<9	1 (reference)	
9–12	1.217 (0.8868 ~ 1.6701)	0.2240
>12	0.7612 (0.5511 ~ 1.0515)	0.0979
Marital status, n (%)		
Married or living with a partner	1 (reference)	
Living alone	1.3975 (1.154 ~ 1.6923)	<0.001
Physical activity, n (%)	0.6933 (0.5408 ~ 0.8889)	0.0038
BMI (kg/m^2^)	0.984 (0.9689 ~ 0.9993)	0.0401
Energy (kcal)	0.9997 (0.9996 ~ 0.9998)	<0.001
Protein (gm)	0.9889 (0.986 ~ 0.9918)	<0.001
Carbohydrate (gm)	0.9986 (0.9977 ~ 0.9994)	<0.001
Total sugars (gm)	1.0001 (0.9989 ~ 1.0013)	0.8520
Dietary fiber (gm)	0.9475 (0.935 ~ 0.9602)	<0.001
Total fat (gm)	0.9951 (0.9927 ~ 0.9975)	<0.001
Coffee (mg)	0.9996 (0.9991 ~ 1.0001)	0.1082
Alcohol (gm)	0.9838 (0.977 ~ 0.9907)	<0.001
Moisture (gm)	0.9997 (0.9996 ~ 0.9998)	<0.001
Dietary supplements, n (%)	0.7105 (0.5856 ~ 0.8622)	<0.001
Diabetes, n (%)	1.061 (0.7814 ~ 1.4405)	0.7044
Coronary heart disease, n (%)	0.9108 (0.548 ~ 1.5139)	0.7186
Stroke, n (%)	1.8805 (1.2386 ~ 2.8549)	0.0030

Data presented are ORs and 95% CIs.

**Table 3:** Association of serum cotinine level, smoking status, and constipation.

Serum cotinine level	Cases/participants	Non-adjusted model		Model 1		Model 2		Model 3	
		OR (95% CI)	*p*-value	OR (95% CI)	*p*-value	OR (95% CI)	*p*-value	OR (95% CI)	*p*-value
Log2-transformed cotinine, ng/mL	440/11651	1.03 (1.02 ~ 1.05)	<0.001	1.02 (1.00 ~ 1.03)	0.106	1.01 (1.00 ~ 1.03)	0.15	1.01 (0.99 ~ 1.03)	0.412
Cotinine categories									
<0.05 ng/mL	162/5607	1.00 (reference)		1.00 (reference)		1.00 (reference)		1.00 (reference)	
0.05–2.99 ng/mL	138/2871	1.70 (1.35 ~ 2.14)	<0.001	1.45 (1.13 ~ 1.85)	0.003	1.44 (1.12 ~ 1.83)	0.004	1.36 (1.06 ~ 1.74)	0.016
≥3 ng/mL	140/3173	1.55 (1.23 ~ 1.95)	<0.001	1.24 (0.96 ~ 1.6)	0.101	1.21 (0.94 ~ 1.57)	0.139	1.13 (0.86 ~ 1.48)	0.379
Trend test			<0.001		0.077		0.109		0.301
Self-reported smoking status, %									
Never	230/6187	1.00 (reference)		1.00 (reference)		1.00 (reference)		1.00 (reference)	
Former	88/2914	0.81 (0.63 ~ 1.04)	0.091	1.18 (0.91 ~ 1.53)	0.223	1.16 (0.89 ~ 1.51)	0.268	1.19 (0.91 ~ 1.55)	0.201
Current	122/2550	1.3 (1.04 ~ 1.63)	0.021	1.19 (0.93 ~ 1.51)	0.16	1.16 (0.91 ~ 1.48)	0.218	1.13 (0.87 ~ 1.46)	0.356
Trend test			<0.078		0.127		0.179		0.272

Model 1: adjusted for age, sex, ethnicity (non-Hispanic white, non-Hispanic black, Mexican American, or other ethnicities), educational attainment (less than 9 years, 9–12 years, and more than 12 years), physical activity, marital status (married, living with a partner, or living alone), and BMI (continuous).

Model 2: adjusted for Model 1 + previous disease (diabetes, stroke, and coronary heart disease).

Model 3: adjusted for Model 2 + additional adjustments for dietary factors (calorie consumption, protein consumption, carbohydrate consumption, sugar consumption, fiber consumption, fat consumption, caffeine consumption, alcohol consumption, and water (moisture) consumption), and dietary supplements.

**Figure 2:**
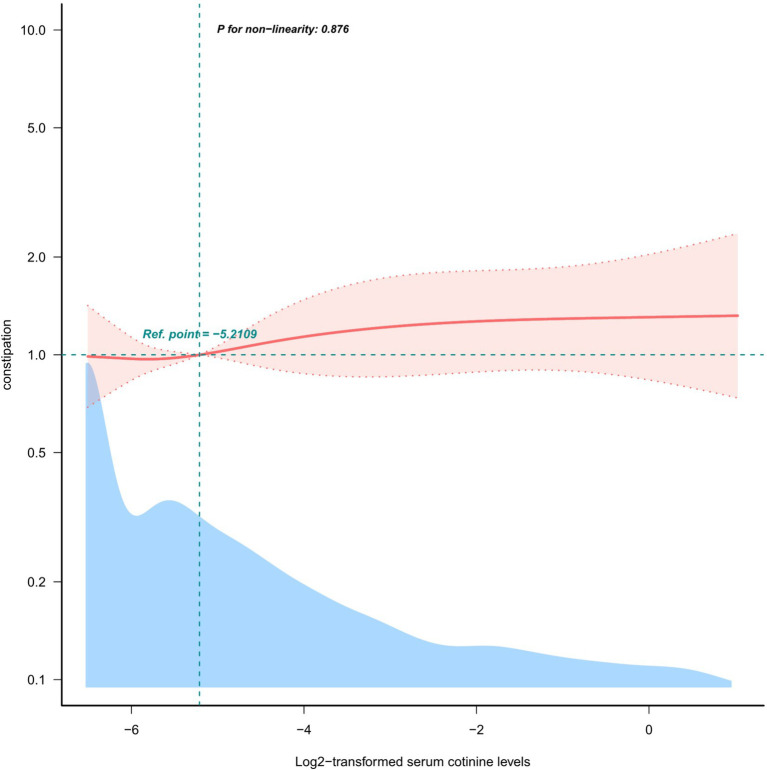
Restricted cubic spline model of the odds ratios of log2-transformed serum cotinine levels below 3 ng/mL with the prevalence of constipation.

To determine the relationship between log2-transformed serum cotinine levels and constipation, restricted cubic spine regression with multivariable adjustment was used. There was a linear and positive association between log2-transformed serum cotinine levels and constipation risk (P for non-linear = 0.876) after adjusting all confounding factors, as shown in Figure 2.

**Figure 3:**
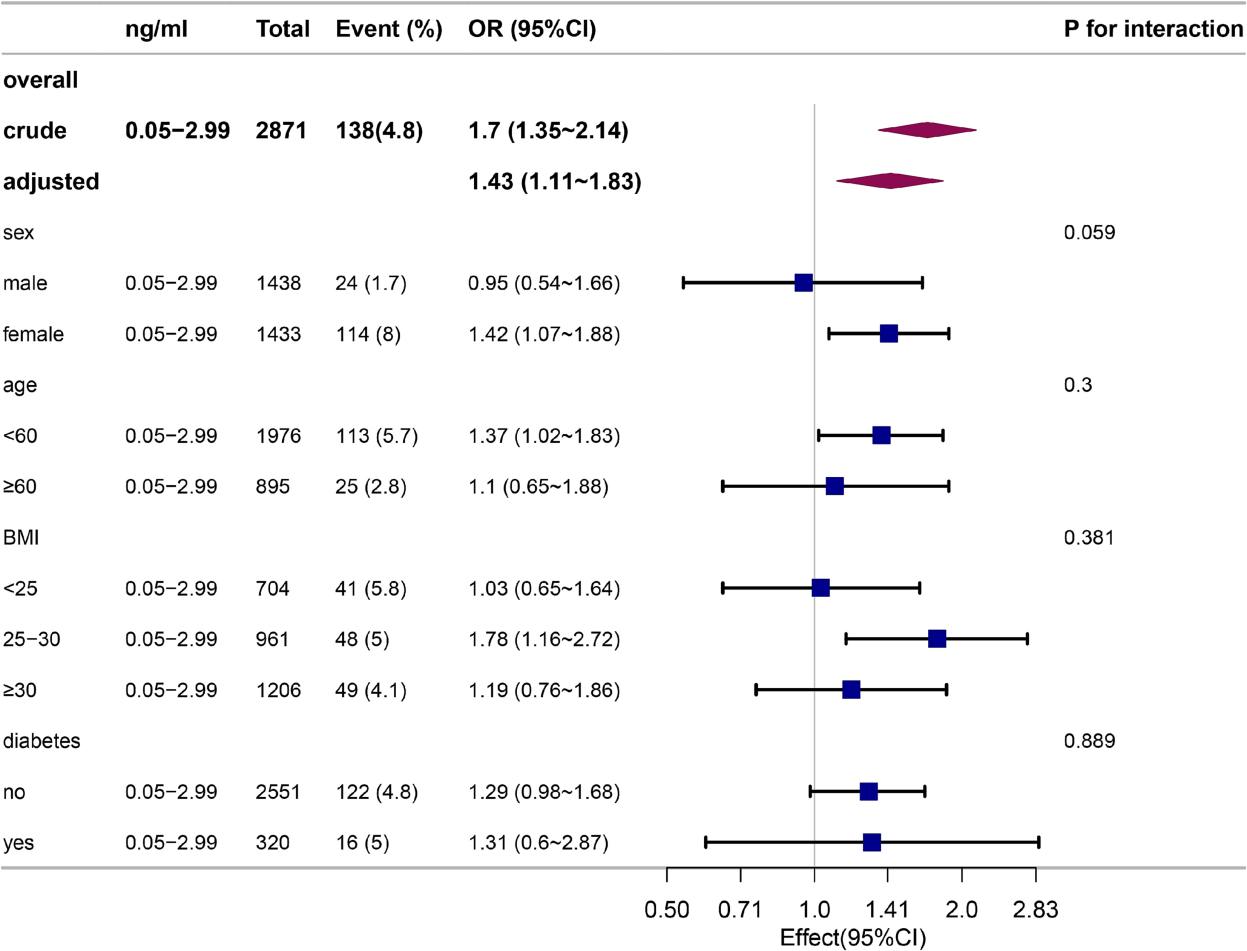
Stratification analysis on the association between participants with cotinine levels of 0.05–2.99 ng/mL and constipation defined by stool frequency in non-smokers.

The data are presented as a subgroup analysis in Figure 3. Subgroup analyses by age, sex, BMI, and previous diseases illustrated no statistically significant interactions (*p* > 0.05) in participants with cotinine levels of 0.05–2.99 ng/mL. In every subgroup, the association between smoke exposure and constipation was relatively stable.

## Discussion

Our findings show that constipation is not influenced by serum cotinine levels or self-reported smoking habits. Even after adjusting for factors such as age, sex, ethnicity, education level, marital status, physical activity, BMI, dietary factors, and previous diseases (diabetes, stroke, or coronary heart disease), this relationship remained constant. In participants with serum cotinine levels between 0.05 and 2.99 ng/mL, fecal frequency-related constipation was positively associated with serum cotinine after adjusting for confounders. However, no such association was found in participants with other cotinine levels. A subgroup analysis illustrated that the association between smoke exposure and constipation was relatively stable in every subgroup.

We found that associations between smoking and constipation, as defined by stool frequency, were consistent with another study (30). However, some studies have shown that smoking is not associated with constipation, bowel frequency, or stool consistency (31, 32). There have been studies on passive smoking and constipation among children as well (33). A new perspective on constipation is provided by our study of serum cotinine levels, which are often used as a means of distinguishing smokers from non-smokers at the cutpoint of 3 ng/mL, and the promoting effect on stool frequency-related constipation may not appear in the general population but may appear when cotinine levels range between 0.05 and 2.99 ng/mL.

As a result of chronic smoking, there is evidence that the gastrointestinal tract undergoes inflammation, resulting in angiogenesis and epithelial dysfunction (34). Tobacco smoke condensate induced antimicrobial peptide production in the Paneth cells and decreased bactericidal activity, making mice more susceptible to bacterial infection and leading to inflammation in the ileum (35). Finally, smoke contains free radicals that induce oxidative stress, impairing antioxidant enzyme activity and causing an imbalance between oxidants and antioxidants; during this disruption, tight junctions within the digestive tract are disrupted, thus increasing constipation to some extent.

Our in-depth research on the correlation between smoking and constipation shows that passive smokers also suffer from constipation. This may be partly due to the effects of nicotine, which increases inflammation, disrupts the antioxidant balance in the body, increases malondialdehyde levels, and reduces superoxide dismutase and catalase production. These factors may lead to damage to the intestinal barrier (36, 37). This finding is consistent with a previous study (7) and a more recent one (38). Constipation is still a serious challenge, so this study is of great interest; avoiding passive smoking can help mitigate the effects of smoke exposure on constipation, as well as prevent and treat constipation.

Standardized, uniform protocols were used to collect and screen the NHANES data to ensure accuracy, consistency, and reliability. As a result of the large sample size of the community, the results were highly reliable. The results of a regression analysis, which accounted for important confounding factors, were more intuitive and comprehensive than those of a mechanism analysis.

Despite this, some limitations remain in the study. Above all, since this study relied on the cross-sectional data from the NHANES, causal relationships could not be implied between dependent variables, independent variables, and covariates. In future studies, we will explore the use of Mendelian randomization to assess the causal relationship between tobacco exposure and constipation. We also plan to conduct a longitudinal cohort study to fully understand the temporal dynamics of the effects of tobacco use on constipation. Moreover, the Rome IV standard defines chronic constipation as a condition characterized by several other symptoms in addition to constipation’s frequency. Therefore, there is no accurate representation of the prevalence of constipation in the current study. Last but not least, the conclusions drawn from the United States may not apply to other populations due to the differences in genetic background, metabolic factors, food habits, and levels of exposure to environmental tobacco smoke (ETS).

## Data Availability

The datasets presented in this study can be found in online repositories. The names of the repository/repositories and accession number(s) can be found at: http://www.cdc.gov/nchs/nhanes.htm.

## Footnotes

^1^http://www.cdc.gov/nchs/nhanes.htm, accessed on 7 March 2024.

^2^ http://www.R-project.org
