# Supplementary material for: Association between tobacco smoke exposure and constipation among American adults: a National Health and Nutrition Examination Survey
Source: Front Public Health. 2025 Mar 4;13:1502341. doi: 10.3389/fpubh.2025.1502341 (PMC11920834; doi:10.3389/fpubh.2025.1502341)
Supplement: Supplementary file 1 [file Data_Sheet_1.docx]

**Supplementary Table 1 characteristics of of excluded and included samples participants**

| Variables | Total (n = 17132) | inluded (n = 11651) | excluded (n = 5481) | *P*-value | statistic |
| --- | --- | --- | --- | --- | --- |
| **Gender, n (%)** |  |  |  | 0.655 | 0.199 |
| Male | 8303 (48.5) | 5662 (48.6) | 2642 (48.2) |  |  |
| Female | 8829 (51.5) | 5989 (51.4) | 2839 (51.8) |  |  |
| **Age (years)** | 49.6 ± 18.3 | 49.5 ± 18.2 | 50.0 ± 18.5 | 0.092 | 2.847 |
| **Race/ethnicity, n (%)** |  |  |  | < 0.001 | 1179.364 |
| Non-Hispanic white | 8232 (48.1) | 6338 (54.4) | 2012(36.7) |  |  |
| Non-Hispanic black | 3472 (20.3) | 2330 (20) | 1140 (20.8) |  |  |
| Mexican American | 3176 (18.5) | 2190 (18.8) | 992 (18.1) |  |  |
| Others | 2252 (13.1) | 803 (6.9) | 1337 (24.4) |  |  |
| **Education level (year), n (%)** |  |  |  | < 0.001 | 159.791 |
| ＜9 | 2210 (12.9) | 1247 (10.7) | 926 (16.9) |  |  |
| 9–12 | 6890 (40.3) | 4660 (40) | 2231 (40.7) |  |  |
| ＞12 | 8001 (46.8) | 5744 (49.3) | 2324 (42.3) |  |  |
| **Marital status, n (%)** |  |  |  | < 0.001 | 57.416 |
| Married or living with a partner | 10281 (60.1) | 7247 (62.2) | 3086 (56.3) |  |  |
| Living alone | 6836 (39.9) | 4404 (37.8) | 2395 (43.7) |  |  |
| **Physical activity, n (%)** | 3764 (22.3) | 2715 (23.3) | 1118 (20.4) | < 0.001 | 17.668 |
| BMXBMI.x, Mean ± SD | 29.0 ± 6.8 | 29.0 ± 6.7 | 29.0 ± 6.9 | 0.783 | 0.076 |
| **Dietary factors** |  |  |  |  |  |
| Energy (kcal) | 2092.7 ± 1000.0 | 2095.5 ± 1008.0 | 2086.1 ± 981.3 | 0.588 | 0.293 |
| Protein (gm) | 80.5 ± 42.4 | 80.7 ± 42.7 | 80.0 ± 41.7 | 0.353 | 0.863 |
| Carbohydrate (gm) | 255.5 ± 126.9 | 255.3 ± 128.0 | 256.0 ± 124.5 | 0.757 | 0.095 |
| Fiber (gm) | 16.0 ± 9.8 | 16.0 ± 9.9 | 16.0 ± 9.8 | 0.745 | 0.106 |
| Moisture (gm) | 2862.2 ± 1476.0 | 2859.9 ± 1472.1 | 2867.6 ± 1485.0 | 0.763 | 0.091 |
| Dietary supplements, n (%) | 8133 (47.5) | 5382 (49) | 2751 (44.8) | < 0.001 | 27.987 |
| Sugar (gm) | 100.1 (62.5, 149.7) | 99.6 (62.5, 149.3) | 102.0 (62.1, 150.7) | 0.209 | 1.578 |
| Coffee (mg) | 98.0 (12.0, 213.0) | 98.0 (13.0, 213.0) | 96.0 (12.0, 218.0) | 0.884 | 0.021 |
| Alcohol (gm) | 0.0 (0.0, 0.1) | 0.0 (0.0, 0.1) | 0.0 (0.0, 0.1) | 0.965 | 0.002 |
| Fat (gm) | 69.2 (45.8, 100.1) | 69.3 (45.9, 100.4) | 69.1 (45.7, 99.2) | 0.524 | 0.406 |
|  |  |  |  |  |  |

**Supplementary Table 2 Association of serum cotinine level, smoking status and constipation defined by stool consistency**

| serum cotinine level | Cases/participants | Non-adjusted Model | |  | Model 1 | |  | Model 2 | |  | Model 3 | |
| --- | --- | --- | --- | --- | --- | --- | --- | --- | --- | --- | --- | --- |
|  |  | OR (95% CI) | *P*-value |  | OR (95% CI) | *P*-value |  | OR (95% CI) | *P*-value |  | OR (95% CI) | *P*-value |
| **Log2-transformed cotinine, ng/ml** | 973/11651 | 1.01 (1.00~1.04) | 0.076 |  | 1.01 (1.00~1.03) | 0.104 |  | 1.00 (1.00~1.03) | 0.163 |  | 1.00 (0.99~1.03) | 0.274 |
| **Cotinine categories** | | | | | | | | | | | | |
| <0.05ng/ml | 476/5607 | 1.00  (reference) |  |  | 1.00  (reference) |  |  | 1.00  (reference) |  |  | 1.00  (reference) |  |
| 0.05-2.99ng/ml | 269/2871 | 1.61 (1.20~2.13) | <0.001 |  | 1.36 (1.08~1.79) | 0.008 |  | 1.41 (1.02~1.78) | 0.005 |  | 1.38 (1.01~1.68) | 0.053 |
| ≥3ng/ml | 228/3173 | 1.43 (1.17~2.01) | 0.031 |  | 1.19 (1.00~1.73) | 0.126 |  | 1.15 (1.00~1.62) | 0.234 |  | 1.07 (0.98~1.53) | 0.426 |
| Trend test |  |  | 0.076 |  |  | 0.113 |  |  | 0.211 |  |  | 0.314 |
| **Self-reported smoking status, %** | | | | | | | | | | | | |
| Never | 582/6187 | 1.00  (reference) |  |  | 1.00  (reference) |  |  | 1.00  (reference) |  |  | 1.00  (reference) |  |
| Former | 205/2914 | 0.73 (0.62~0.86) | <0.001 |  | 0.95 (0.8~1.13) | 0.575 |  | 0.94 (0.79~1.12) | 0.518 |  | 0.97 (0.81~1.16) | 0.742 |
| Current | 186/2550 | 0.76 (0.64~0.99) | 0.052 |  | 0.76 (0.62~0.99) | 0.051 |  | 0.79 (0.61~1.01) | 0.301 |  | 0.74 (0.59~1.00) | 0.403 |
| Trend test |  |  | <0.001 |  |  | 0.062 |  |  | 0.102 |  |  | 0.205 |

**Supplementary Table 3 Subgroup analyses based on self-reported smoking status in passive smokers**

| Subgroup | n.total | n.event_% | crude.OR_95CI | crude.P_value | adj.OR_95CI | adj.P_value | P.for.interaction |
| --- | --- | --- | --- | --- | --- | --- | --- |
| Never | 5808 | 214 (3.7) | 1.86 (1.42~2.44) | <0.001 | 1.46 (1.06~2) | 0.021 | 0.850 |
| Former | 2555 | 80 (3.1) | 1.55 (1.02~2.38) | 0.042 | 1.43 (0.88~2.32) | 0.154 |  |
| Current | 115 | 6 (5.2) | 1.35 (0.49~3.69) | 0.563 | 0.05 (0~Inf) | 1 |  |
